# Deaf role-models for Deaf children in hearing families: a scoping review

**DOI:** 10.1093/jdsade/enae028

**Published:** 2024-08-11

**Authors:** Angela Joy, Susan Ledger, Jill Duncan

**Affiliations:** School of Education, College of Human and Social Futures, University of Newcastle, Callaghan, Australia; School of Education, College of Human and Social Futures, University of Newcastle, Callaghan, Australia; School of Education, College of Human and Social Futures, University of Newcastle, Callaghan, Australia

## Abstract

The use of Deaf role-models (DRMs) with Deaf children born into hearing families is a practice aimed at improving outcomes for Deaf children, yet there is little peer-reviewed research available to influence future direction of such. This scoping review directs attention to available research on DRMs as a socio-linguistic and cultural viewpoint for balancing a predominantly audiological approach for early intervention for Deaf children. Systematic database searches initially yielded 132 records, of which seven articles were included in this scoping review. Findings are presented as five themes: ‘Deaf Gain’ and associated cultural capital, effective communication, developmental influences, family (or caregiver) attitudes to Deafness, and administration of DRM programs. Few formalized DRM programs were identified within the literature. The review concludes with recommendations for further exploration of the DRM experiences of Deaf people and their families within Australia.

Deaf^1^ role-models (DRMs) are Deaf adults who provide mentoring to Deaf children and their hearing family members by sharing their lived experience of Deafness to support the development of the Deaf children with whom they work (Gale et al., 2021; Giaouri et al., 2022; Rogers & Young, 2011; Shuler-Krause & White, 2019). DRMs may perform a formally recognized role as a part of an official role-model or mentor program, for which they have received specific role-model training, or their role-model status may be perceived or attributed to them informally through circumstances such as their professional role of child care workers, teachers or advocates for Deaf children (Fobi et al., 2022; Gale et al., 2021; Giaouri et al., 2022; Golos et al., 2018; Rogers & Young, 2011; Shuler-Krause & White, 2019). This scoping review specifically focuses on DRMs in the early years between identifying Deafness and the commencement of formal schooling.

Seminal mentor training programs, such as the Deaf Mentor Experimental Project (Watkins et al., 1998) have inspired more recent attention to the role that DRMs can play in contributing to Deaf children making significant gains in language and communication skills (Fobi et al., 2022; Hamilton & Clark, 2020), and in the social–emotional, identity and academic development of Deaf children (Hamilton & Clark, 2020). Raj and Pitchai (2015) have identified domains that are affected by mild or greater hearing loss which can influence one’s “Quality of Life” (QoL). These domains are educational implications, social integration, social–emotional well-being, speech, language and communication across both spoken and signed modalities, family relationships, leisure time activities, and general functioning. Improved QoL when young children are connected with DRMs is referred to in recent research as another reported benefit for families (Fobi et al., 2022; Petersen et al., 2016).

For Deaf children born into hearing families, the language that they are initially exposed to is that of their hearing parents, the modality is spoken, and they are surrounded by the culture of the family into which they are born. For Deaf children born into signing Deaf families, their first language utilizes a visual/spatial modality and is intertwined with Deaf culture. The difference between these family contexts is the level of accessibility that the Deaf child has to the language of their parents. Not having immediate access to a first language (i.e., not being able to hear the spoken language of the parents) can pose serious consequences that impact upon the child’s QoL, including delayed language development, educational ramifications such as academic underachievement, and mental health concerns connected with social isolation (Hall, 2017; World Health Organization, 2018). Whilst early identification of hearing loss and the fitting of hearing aids and/or cochlear implantation has resulted in improved literacy outcomes for Deaf children (Pimperton et al., 2016), a large proportion of these children still experience delays, particularly regarding language and social–emotional development (Clark et al., 2020; Holzinger et al., 2022), which have been attributed to a negative influence on their QoL (Raj & Pitchai, 2015; Ronner et al., 2020). Providing access to a signed language from the time of the identification of Deafness has been highlighted as a valuable option for supporting communication and language development, potentially mitigating the negative effects of delayed auditory access (Hall et al., 2017; Hamilton & Clark, 2020).

The purpose of this scoping review was to identify what is known about the influence of Deaf role-models for Deaf children born into hearing families, and to collate these learnings into themes that potentially will guide future early intervention practice and policy for young Deaf children. Providing a synthesis of current research about DRMs is essential in offering a cultural-linguistic adjunct to the predominant audiological interventions for young Deaf children.

## Positionality Statement

The first author is a doctoral candidate and a Teacher of the Deaf. The second author is a Dean of Education and co-supervisor. The third author is also a Teacher of the Deaf, has had permanent conductive hearing loss since adolescence, and is the candidate’s principal supervisor.

## Method

A scoping review was chosen in preference over a systematic review as being most appropriate for this investigation due to the nature of the research question. Exploring what is known can inform future research and policy development by exposing gaps in the current literature (Tricco et al., 2018). The objectives of this scoping review were to explore existing global peer-reviewed research about DRMs being utilized as an early intervention for Deaf children born into hearing families during the years before school begins, and to identify common conclusions or gaps in knowledge that are pertinent within an Australian context.

A limitation of scoping reviews has been observed around methodological quality and reporting being inconsistent (Tricco et al., 2018). The Preferred Reporting Items for Systematic Reviews and Meta-Analysis (PRISMA) has therefore been extended to guide scoping reviews. Peters et al. (2020) have updated methodological guidance for the conduct of scoping reviews with support from the Joanna Briggs Institute. The PRISMA-ScR checklist (Tricco et al., 2018) has therefore been used to form the basis of this scoping review.

### Research Question

The research question explored was: What is known about the influence of DRMs in early intervention settings for Deaf children born into hearing families? This question endeavored to explore studies that included the perspectives of DRMs, Deaf people who received an early intervention that included Deaf mentors such as DRMs, and hearing family members of Deaf children.

### Search Strategy

Systematic electronic searches were performed in March and April of 2023 to locate academic articles related to the topic using EBSCO Megafile Ultimate, ProQuest, Sage, and Scopus. Search terms used were as follows:

1. (Deaf OR “hear^*^ impair^*^” OR “hard of hearing”) AND (“role model^*^” OR mentor^*^) AND “early intervention”
2. (“Deaf role model^*^” OR “Deaf mentor^*^”) AND (“famil^*^ OR child^*^”)

### Inclusion Criteria

The included studies met four criteria. First, articles published post-2000 were selected for data collection as this was the year that the first large-scale newborn hearing screening program in Australia was established, following the inception of similar programs across the U.S. and the U.K. (Coates & Gifkins, 2003). Post the 2000 onset of newborn infant hearing screening, early technological and language interventions (including the potential for the introduction to a DRM) could begin in the first few months of life (Ching et al., 2017; Yoshinaga-Itano et al., 2021). Second, peer-reviewed empirical research articles were sought when searching for articles on the databases, thus literature reviews and government or organizational commissioned documents were thereby not included. Third, reviewed articles all focused on early intervention mentoring post-identification, in the early education years before formal schooling begins, by Deaf people who are bimodal/bilingual/bicultural or by Deaf people who focus primarily on listening and spoken language development. Fourth, articles that investigated mentors or role-models who were Deaf themselves—not parent role-models (other hearing parents of Deaf children providing support to parents) were selected.

## Results

The Appendix identifies the reviewed articles and labels the extracted data in categories outlining the country in which the research took place, the number of participants involved, the study design, a summary of key findings, and recommendations based on the findings. The seven selected articles (see Appendix), highlight some common understandings regarding what is known globally about the influence of Deaf role-models (DRMs) on Deaf children born into hearing families. In all, the data included the responses of close to 1,000 Deaf and hearing participants. These included parents and other family members, educators and coordinators of educational settings, audiologists, speech therapists, Deaf role-models, and other professional practitioners. Whilst this scoping review aimed to include studies that explored the perspectives of DRMs, Deaf people who received an early intervention that included Deaf mentors such as DRMs, and hearing family members of Deaf children, the perspectives of other professionals such as educators, audiologists and speech therapists could not be dismissed as they were also captured in the reviewed articles. They provided data about their observations of Deaf adults working with children in early intervention programs. The data came from both qualitative (n = 3), quantitative (n = 1), and mixed method (n = 3) study designs which had a range of study purposes.

## Data Extraction

**Figure 1:**
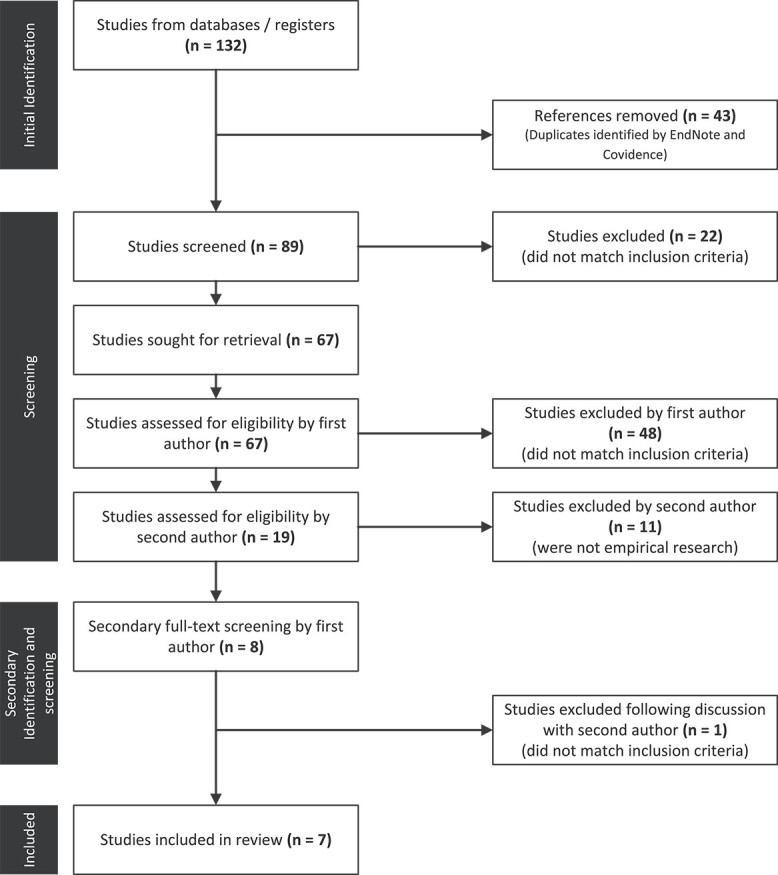
Flowchart illustrating search and selection process.

As illustrated in Figure 1, the database searches produced 132 results. Upon removing duplicates, 89 remained. The first author reviewed the titles and abstracts of these articles and excluded 22 that did not meet the inclusion criteria. Sixty-seven full-text studies were assessed for eligibility and a further 48 were excluded for not meeting the inclusion criteria. The third author screened the remaining 19 studies for interrater reliability. It was agreed to only include empirical research papers, not professional opinion pieces or literature reviews, and 11 articles were consequently excluded. One additional article was later omitted following discussion as its focus was on mentoring during the years of schooling and work placement rather than during the early intervention years before the commencement of school. Regular discussions between all authors took place to ensure all were in agreeance with the decisions regarding the inclusion or exclusion of articles. This resulted in seven included articles.

### Data Analysis

**Figure 2:**
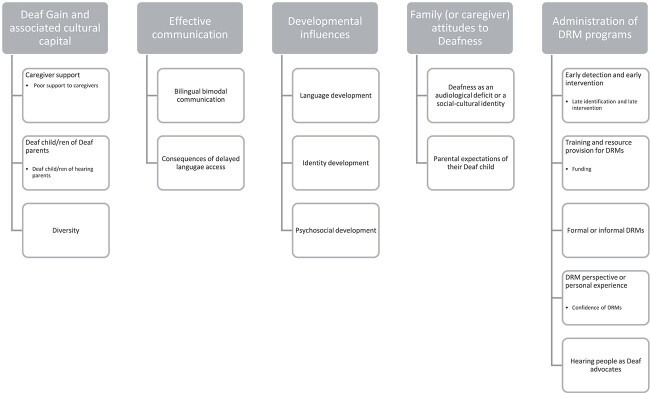
Hierarchy of themes determined by thematic analysis.

The first author undertook a thematic analysis of the seven selected articles using NVivo 12 qualitative research software (QRS International, 2018) to identify the key themes that were relevant to the research question. This was undertaken in line with Braun et al.’s (2019) thematic approach to analyzing patterns of themes that the first author identified. Twenty-five individual codes were created whilst analyzing the seven articles, with a total of 431 highlighted passages. Those codes were organized into five overarching themes by developing a hierarchy using the “parent node” option in NVivo. See Figure 2 for theme hierarchy.

Five main themes were identified in all seven articles: (1) “Deaf Gain” and associated cultural capital, (2) effective communication, (3) developmental influences, (4) family (or caregiver) attitudes to Deafness, and (5) administration of DRM programs. Descriptions of these themes and associated sub-themes are presented within the discussion section.

## Discussion

This scoping review aimed to explore existing global peer-reviewed research about DRMs being utilized as an early intervention for Deaf children born into hearing families, and to identify common themes. Subsequently, the authors were able to apply literature-based information to an Australian context.

The review process identified the following five major themes (see Figure 2).

### “Deaf Gain” and Associated Cultural Capital

*Deaf Gain* is an affirming concept devised by Bauman and Murray (2014) that emphasizes the unique benefits of Deafness, challenging the polarized view of Deafness as a disability. Hamilton and Clark (2020) also use this term as an antonym for *hearing loss*, referring to Deaf people as having a “cultural and linguistic difference” rather than being “defective, and needing to be fixed” (p. 714). They liken this perspective to the phrase “Deaf way of knowing” (p. 715), which is similar to the cultural capital, or innate positive resources that Deaf people have due to their lived experience of Deafness. Cultural capital relates to language, aspirations, social or community connections, navigating life, and having a strong sense of self (Gale et al., 2021; Giaouri et al., 2022; Golos et al., 2018; Hamilton & Clark, 2020; Rogers & Young, 2011). Articles that used similar terminology such as “cultural wealth”, “Deaf-centric knowledge” (Hamilton & Clark, 2020), “shared life experiences” (Shuler-Krause & White, 2019), or similar, were also coded with this overarching theme.

The sub-theme of diversity referred to parts of text highlighting how Deaf people are not a homogenous group. The “cultural and linguistic difference” that Hamilton and Clark (2020, p. 714) refer to can be extended to include the diversity in cultures, language, and life experiences that Deaf people can share with others. There are a multitude of various languages used by Deaf people. For example, Fobi et al. (2022) mention in their African study that there are more than seventy-nine spoken languages and three sign languages in Ghana. Gale et al. (2021) noted that Deaf adults occupy a diverse range of roles within early intervention settings, which shows hearing parents that Deaf people can and do have diverse expertise and professions.

Deaf adults exhibit a wide range of life experiences that vary based on their ethnicity, language, lifestyle, community, and cultural connections, gender, hearing level, use of technology, education levels, gender, health, ability status, etc (Gale et al., 2021; Giaouri et al., 2022; Golos et al., 2018; Rogers & Young, 2011). Giaouri et al. (2022) noted in their research that despite the need for these kinds of multidimensional affiliations, families often are not exposed to a diverse range of DRMs in early intervention programs, and subsequently, many Deaf children do not have a DRM that shares their life experience. However, in the DRM project that Rogers and Young (2011) evaluated over a decade earlier, having a diverse range of DRMs to choose from was an aim of the project that was successfully met, suggesting that it is not only a desirable objective but also entirely possible. Golos et al. (2018) discuss the importance of supporting Deaf students in early childhood settings with a diverse range of live and symbolic (in books and other media) DRMs to counter the fact that despite the diversity in backgrounds of Deaf children, most of their teachers are white, hearing teachers. Hamilton and Clark (2020) admit that one of the limitations of their research was they did not interview racially diverse families.

Sub-themes that examined the relationship between Deaf children and Deaf parents, and Deaf children of hearing parents were also investigated. Gale et al. (2021) note that the developmental outcomes of Deaf children with Deaf parents are higher than Deaf children who have hearing parents. Subsequently, they express their opinion to justify the role of DRMs. “It is important to infuse Deaf adults in early intervention because more than 90% of Deaf children are born to hearing parents and the parents may have little or no experience with Deaf people or even expectations regarding their Deaf child” (Gale et al., 2021, p. 18).

Caregiver support and consequences of poor support provided to caregivers were also included under this theme, with Fobi et al. (2022) outlining how the support provided by DRMs can align with many of the *Deaf Gain* and cultural capital ways of helping a young Deaf child navigate their life, and also includes love, warmth and emotional support to parents and caregivers as well as practical resources such as sign language dictionaries, phone apps, and logistical guidance about available services from other support or educational organizations. They, along with Hamilton and Clark (2020), claim that families without DRM support are at risk of polarized perspectives of Deafness that risk their child having delayed language access and are subsequently at risk of language deprivation, as well as academic and social impairments.

### Effective Communication

Effective communication included references to accessible language, and sub-themes included bilingual bimodal communication and consequences of delayed language access. Bilingual bimodalism refers to using or learning two separate languages, one spoken and one signed as an adjunct to each other as an intervention to address the potential risk of language deprivation or delay from a spoken language only approach (Hamilton & Clark, 2020). Fobi et al. (2022) interviewed Deaf adults regarding their perceptions of the role of DRMs in early childhood education settings for Deaf children and noted that there are concerns about facilitating communication between Deaf children and the adults in their lives, both at home and in the community. They stress that it is not the Deafness of the child that is of concern, but rather it is the level of language and communication that both parties (adults and children) have to establish an effective relationship between the child and caregiver. Two of their 17 participants indicated that the “bond between caregivers and their children depends on the communication abilities of both” (p. 4). This parallels Shuler–Krause and White’s (2019) findings that when DRMs provided information about accessible communication, this subsequently assisted families in developing their confidence in being able to communicate with their Deaf child.

For communication to be effective, participants require access to the language, and as Giaouri et al. (2022) explain, most hearing parents rely on spoken language to communicate with their Deaf child, which the Deaf child may or may not be able to access with hearing aids and/or cochlear implants (Hamilton & Clark, 2020). Conversely, sign languages are visual and do not pose barriers to acquisition for most Deaf children. Hamilton and Clark (2020) and Fobi et al. (2022) iterate the message that sign languages do not need to be avoided if a family’s goal for their child is communication via spoken language; signed and spoken language development can develop in parallel, thus avoiding the barriers to effective communication.

Most of the reviewed articles list academic delay, academic underachievement, social isolation, and social–emotional impairments as the major consequences of delayed language acquisition (Fobi et al., 2022; Gale et al., 2021; Giaouri et al., 2022; Golos et al., 2018; Hamilton & Clark, 2020). Giaouri et al. (2022) discuss DRMs providing services to families using both the spoken language of the family, and the signed language of the local Deaf community, resulting in these children making more language gains than Deaf children who did not have DRMs involved with their families. Golos et al. (2018) discuss the benefits of a language-rich environment supported by bilingual adults which allows an opportunity for Deaf children to be exposed to the phonology of sign language and make connections with the spoken or written language that they are also exposed to in their lives. Golos et al. (2018) link this discussion with their survey finding of DRMs having varied success levels with a child’s literacy development depending upon the sign language ability and communication mode of the DRM and supporting educator. Signing, fingerspelling, and pointing to words in text were all reported approaches to connect the signing with written language utilized by adults in the early intervention settings. Rogers and Young (2011) state that often this approach is not introduced until the years of schooling, yet families who have had a bilingual bimodal DRM in a family-centered early childhood context thrive; a finding similar to that of Hamilton and Clark (2020) almost a decade later. Hamilton and Clark (2020) stress that whilst many Deaf children thrive without sign language, they highlight that many others do not thrive, and it is only once the critical period for language has passed that they are offered signing as a language intervention, based upon their belief that it is an inaccurate premise that if the primary goal of spoken language is not successful then it is not too difficult to learn a sign language later in life.

### Developmental Influences

This theme included sub-themes of language development, identity development, and psychosocial development. The research strongly points to DRMs in the early childhood years providing communication support and language modeling being a key factor in addressing delays in development (Fobi et al., 2022; Gale et al., 2021). Fobi et al. (2022) also stated that parents who had been supported by DRM programs have said that they felt their child had an improved QoL due to their participation. In addition to the language, academic, and social development benefits for the Deaf child, there are positive influences on the self-esteem, confidence, and expectations of the hearing caregivers of young Deaf children (Rogers & Young, 2011) as DRMs support new hearing caregivers along their journey of navigating unexpected new experiences as they care for their Deaf baby (Fobi et al., 2022; Hamilton & Clark, 2020).

Fobi et al. (2022) and Hamilton and Clark (2020), when referencing a seminal DRM project (the Deaf Mentor Experimental Project), commented that their recent investigations concur that Deaf children who had a DRM were reported to have made greater social, academic and language gains than the children who did not. Fobi et al. (2022), after surveying Deaf people involved in early childhood education in Ghana, also stated that parent participants of DRM programs felt their sign language skills, and general ability to effectively communicate with their Deaf children, had improved, and they expressed that their children had improved overall QoL due to their participation in a DRM program.

Giaouri et al. (2022) discuss how language development and identity development can be intertwined, especially when DRMs provide fluent sign language skills along with connection to a local Deaf community; thus, influencing both the language and identity development (encompassing self-esteem and pride in one’s Deafness) of the child. They, along with Golos et al. (2018), assert that the influence of DRMs extends beyond tangible outcomes such as academic achievement, and has a greater contribution to the development of a child’s confidence and self-esteem, which enables a child to more easily navigate a world dominated by hearing people. When discussing the findings of their research, Golos et al. (2018) stress that when Deaf children see an aspect of themselves reflected in others, especially during the important early childhood years, it affirms their reality, leads to a feeling of acceptance, promotes a more positive sense of self and a sense of worthiness in Deaf children.

Shuler-Krause and White (2019) declare that there is widespread agreement about the positive developmental influence of DRMs, however, they acknowledge there is little actual data available on the topic. They recommend that more data be collected as the provision of DRM services grow in order “to ensure that resources are expended in ways that will be most beneficial to families” (Shuler-Krause & White, 2019, p. 61).

### Family (or Caregiver) Attitudes to Deafness

Attitudes towards or about Deafness are often intertwined in the literature with the delineation of Deafness being defined as either an audiological deficit or a social-cultural identity (Giaouri et al., 2022; Golos et al., 2018; Hamilton & Clark, 2020). As Golos et al. (2018) decree, when Deafness is portrayed as a pathological condition, Deaf children (and potentially their caregivers) may feel that it points to a deficiency in the child that needs to be fixed. In contrast, when Deafness is valued as a cultural identity, it can provide a “foundation for resilience” leading to an “increased likelihood for long-term positive outcomes” (p. 43).

Giaouri et al. (2022) discuss the changes that parents have in attitudes toward Deaf people and their culture in response to being exposed to DRMs, such as feeling more able to parent their Deaf children due to reduced feelings of isolation. Learning to sign with their child/ren and seeing improvements in their child’s communication competence because of the encounters they have had with Deaf adults also enables parents to develop a stronger sense of competence in their child’s upbringing (Fobi et al., 2022; Gale et al., 2021; Giaouri et al., 2022).

Gale et al. (2021), along with Rogers and Young (2011) discuss the observance of changes in parental attitudes that the DRMs themselves note. Both articles comment on hearing families with DRM involvement developing a more positive perspective around Deafness, and those parents felt more confident in terms of their outlook for their children. Furthermore, Hamilton and Clark (2020) noted that families who worked with a DRM developed an understanding “that their children were different, but not disabled” (p. 723). The modeling provided by successful Deaf adults was a crucial factor in assuring hearing parents that “their child could grow up to become a productive adult with degrees in higher education and contribute to society as a successfully employed person” (p. 724).

The focus on Deafness as a disability is based on an epistemology that sees Deafness as an audiological deficit that requires fixing (Giaouri et al., 2022; Golos et al., 2018; Hamilton & Clark, 2020). Hamilton and Clark (2020) argue that if Deafness were considered as more of a cultural and linguistic difference, then the terminology of ‘disability’ could only be applied if there is no provision for an accessible and appropriate language, thus the terminology does not apply to the Deaf person, but rather it lies within the culture. As Gale et al. (2021) assert, it can come as a surprise to hearing families that many expectant Deaf parents often hope that their child is also Deaf. Rogers and Young (2011), when reflecting on the perspectives of the DRMs involved with hearing families, revealed that several DRMs were shocked by the initial attitudes they observed in the hearing families. They were surprised by the low expectations that some parents had of their Deaf child, or Deaf people in general, and felt hurt by the realization that some parents felt ashamed of their child’s Deafness. Through these encounters, however, the DRMs realized how important their role was in helping parents realize that their Deaf children could be successful in life. One DRM asserted that it was their role to help parents “to believe in a positive future for their Deaf child” (Rogers & Young, 2011, p. 12).

The reviewed research attests to the importance of hearing families having connections with DRMs to assist with this developing understanding and acceptance of Deafness and Deaf culture, subsequently leading to the hearing family members’ emergent optimistic perspectives and attitudes around both the potential of their Deaf child, and their confidence in parenting them (Fobi et al., 2022; Gale et al., 2021; Giaouri et al., 2022; Golos et al., 2018; Hamilton & Clark, 2020; Rogers & Young, 2011; Shuler-Krause & White, 2019).

### Administration of DRM Programs

Deaf adults perform a crucial role in providing early support for Deaf children and their hearing family members (Fobi et al., 2022; Gale et al., 2021; Giaouri et al., 2022; Golos et al., 2018; Hamilton & Clark, 2020; Rogers & Young, 2011; Shuler-Krause & White, 2019). Gale et al.’s (2021) article highlights “an international consensus of best practices for programmes serving young Deaf children and their families” (p. 3) which was developed by parents and professionals (Deaf and hearing) at a congress called Family-Centred Early Intervention (FCEI). Included in the evidence based FCEI best practice principles was the unequivocal endorsement of incorporating Deaf adults in early intervention programs as part of a professional multidisciplinary team that provides social and emotional as well as practical support to the Deaf child and their families. These principles were influenced by recommendations regarding Deaf people being involved in early intervention programs published by the Joint Committee on Infant Hearing (JCIH) in the U.S., but as Gale et al. (2021) proclaim, they can be extended to early intervention programmes around the world.

Early detection of Deafness through newborn hearing screening programs is the beginning point in the journey for the families of many Deaf children to receive information on the technology and communication support services available to them (Fobi et al., 2022; Gale et al., 2021). While the JCIH recommends that all children be screened before they are a month old, an identification of Deafness be determined before three months and have early intervention established before they are 6 months of age, this often only occurs through the newborn hearing screenings in well-resourced parts of the world while many Deaf children in less-resourced countries are not identified until after twelve months of age (Fobi et al., 2022). Hamilton and Clark (2020) suggested including DRMs in the newborn screening process, especially when presenting information to the family post-identification.

Training of DRMs has been recognized in many of the reviewed articles as a key factor in the success of a DRM program (Fobi et al., 2022; Gale et al., 2021; Giaouri et al., 2022; Hamilton & Clark, 2020; Rogers & Young, 2011; Shuler-Krause & White, 2019). Fobi et al. (2022) stress that academic training, either provided through universities or Deaf associations, can offer capacity building to Deaf adults “that is sensitive of early years development of children who are Deaf” (p. 8). Hamilton and Clark (2020) discuss the level of training that DRMs received that enabled them to deliver the U.S.’s Ski Hi Deaf Mentor curriculum. The DRM project in the United Kingdom offered a similar level of training (Rogers & Young, 2011). Rogers and Young (2011) acknowledged that Deaf people have a significantly higher rate of unemployment than the general population, therefore “there are fewer opportunities for Deaf people to develop their professionalism in comparison with the hearing population” (p. 6). Considering this, the National Deaf Children’s Society in the United Kingdom elected to create a new workforce of DRMs, providing the training and resources themselves. Shuler-Krause and White (2019), in their review of three U.S. surveys regarding DRMs as an early intervention service, mention that recruitment and training of DRMs was a particular area that is seen as a challenge. Across the U.S., many more families require services by DRMs than the available programs can deliver.

As noted above, some DRM programs have embedded training around a particular curriculum, such as the DRM project or the Ski Hi Deaf Mentor curriculum (Hamilton & Clark, 2020; Rogers & Young, 2011). Other researchers (Fobi et al., 2022; Shuler-Krause & White, 2019) mentioned that Deaf children and their families may receive DRM support that is not “systematized and structured” (Fobi et al., 2022, p. 8), or be informal opportunities to interact with Deaf adults (Shuler-Krause & White, 2019). Many DRM interactions and services for providing support to Deaf children and their families are diverse, informal, and individualized without clear infrastructure.

Giaouri et al. (2022) note that despite official DRM programs being in existence for a while, and positive outcomes having been reported for Deaf children and their families, there has been very little attention given to the experiences that the DRMs themselves have had, however Rogers and Young (2011) report that DRMs felt that the families they worked with had a higher positive attitude towards Deafness and there were positive benefits for their children. There is also mention in the reviewed literature of the experience of being a DRM contributing to a sense of worthiness that subsequently increased the personal confidence levels and self-esteem of the DRMs (Rogers & Young, 2011).

Hearing people serving as advocates for the Deaf community was mentioned in the reviewed literature with Golos et al. (2018) acknowledging that there is a place for hearing people to work with young Deaf children, especially if they are fluent in sign language. Whilst they can provide a language model and be an advocate for Deaf children and their families, the specific function of DRMs cannot be replaced by someone without a lived experience of Deafness.

### Applicability of Findings in the Australian Context

Approximately 97% of newborns in Australia are assessed for hearing loss via a Universal Newborn Hearing Screening program (Neumann et al., 2020). Of these, approximately 300 children are identified annually as having a degree of Deafness (Australian Bureau of Statistics, 2022; Department of Education and Early Childhood Development, 2010; Victorian Government, 2021). Additionally, according to Fitzgibbons et al. (2023), the prevalence of Deafness doubles in the period between newborn identification and when children are school-aged. Upon confirmation of a hearing loss, the Australian Government protocols state that families should be provided with “unbiased information on the range of services available, including services provided by Australian Hearing and other early hearing intervention services” (Australian Government Department of Health, 2013, p. 10). At the time of identification, parents must make a series of important and life-changing decisions, including utilization of assistive hearing technology (hearing aids and/or cochlear implants), language modality choice (spoken or signed), and early intervention options (Flaherty, 2015; Stephens & Duncan, 2020, 2022).

Australian researchers have recognized the existence and effects of an unwitting audiological bias despite the post-identification guidelines (Ching et al., 2018). The Australian Government protocols are usually presented by medical professionals, such as doctors, audiologists, and surgeons, who often focus on fixing the hearing deficit and teaching a child to speak. Gale (2021) stresses that in addition to this audiological focus, there ought to be a process that connects the child and their family with Deaf professionals who would be positioned to introduce the family to the language and culture of the Deaf community. For the 90%–95% of families who have no experience of Deafness, and no knowledge of sign language (Mitchell & Karchmer, 2004), the inclusion of DRMs at the point of identification can facilitate a family’s ability to learn to sign (Hamilton & Clark, 2020), thus addressing the existing bias within post-identification policy and practice in Australia. The preference for an audiological focus is not confined to Australia however, with reports of less than 2% of Deaf children worldwide receiving exposure to a signed language during the critical period of language acquisition in their early childhood years (Haualand & Allen, 2009; Murray et al., 2019).

Based on the findings within the literature, the authors can infer that future policy and practice in Australia would benefit from including DRMs as an early intervention approach for Deaf children born into hearing families.

### Summary of Evidence

This scoping review was undertaken to answer the question: What is known about the influence of DRMs in early intervention settings for Deaf children born into hearing families? The literature revealed that Deaf children who have access to DRMs during the early intervention period benefit in terms of their academic, language, and social development (Fobi et al., 2022; Gale et al., 2021). Additionally, there are positive influences for the hearing parents on their self-esteem, confidence, and expectations for their Deaf child (Rogers & Young, 2011), all of which subsequently benefit the child (Fobi et al., 2022). Fobi et al. (2022) declare that when DRMs are involved during early intervention and provide a fluent language model from an early age, “development is positively affected in almost all facets of their lives particularly in areas of communication, socio-emotional, and cognition” (p. 2). This notion was supported by the qualitative evidence presented in their article (see Appendix).

Of the established and formalized DRM programs worldwide, the reviewed articles highlighted the “Deaf Mentor curriculum” that was developed by the Ski HI Institute and the “Guide By Your Side” program used by Hands and Voices, both in the USA (Shuler-Krause & White, 2019). Other reviewed studies discussed the role of DRMs in a more informalized manner, in which the DRM may have been a sign language teacher, counselor, early intervention provider or a mentor with no specific training in a particular formalized DRM curriculum (Fobi et al., 2022; Gale et al., 2021; Giaouri et al., 2022; Golos et al., 2018; Shuler-Krause & White, 2019).

Two of the reviewed studies explored the experiences and perceptions of the DRMs themselves who were involved in the DRM Project in the United Kingdom (Rogers & Young, 2011) and in the early care and education of young Deaf children in Ghana (Fobi et al., 2022). Hamilton and Clark (2020) noted that the Rogers and Young (2011) study was one of only two that had previously evaluated the influence of DRMs, the other study being an evaluation of the Deaf Mentor Experimental Project (Watkins et al., 1998), which was not included in this review due to it having occurred 25 years ago, and thus outside of the criteria for inclusion. One additional DRM program (the Canadian Counselling and Home Training Program) had been found in even earlier research (Greenberg, 1983) and thus was also excluded from this review due to being published before 2000, which was the timeframe established for this study.

In addition to the formalized projects mentioned above, two other formalized programs exist—the First Signs service in New Zealand and Life Track’s Deaf Mentor Family Program in the USA. However, articles that discussed these were not included in this scoping review because their evaluation documents were commissioned either by the government or the organization themselves, with no empirical data being available (Ministry of Education, 2019; Petersen et al., 2016).

The reviewed literature highlights that there has been a revisited interest in seminal DRM programs, with a particular focus being on the theme of Deaf Gain and associated cultural capital. ‘Deaf Gain’ relates to focusing on the positive contributions that Deaf people bring to society rather than on the loss of an audiological function (i.e., ‘hearing loss’), with an emphasis on the perceptions, perspectives, and insights that many hearing people are not aware of (Bauman & Murray, 2014), and cultural capital is defined as innate resources that a person has (or group of people have) that can help others improve their life (Yosso, 2005). DRMs primarily sharing their skills around effective communication, whether that are by way of signed or spoken language, have been conducive to the provision of favorable influences for both Deaf children and their hearing caregivers.

### Limitations and Recommendations

Whilst much of the global research is transmittable across the world, more current study is needed in the Australian context, especially with a focus on Deafness as a cultural and linguistic identity. Australian data on the reported influences was minuscule within the literature, and evidence of the existence of formal role-model programs within Australia was non-existent. It is therefore recommended that further research explore the experiences that Deaf children and their hearing families have had with informal role-model programs or unofficial mentors in Australia and connect this with the international experience that has been discussed within the literature.

Of the seven articles reviewed, six were published within the last 5 years, indicating that there is renewed attention to programs involving DRMs. There is a noticeable gap in the literature of empirical research undertaken in the last decade that evaluates specific DRM programs from a family perspective, and from the reflective perspective of Deaf children who received a DRM as an early intervention service. This gap emphasizes a need for current research about the influence of DRMs on Deaf children born to hearing families.

## Conclusion

The research question guiding this scoping review asked what is known about the influence of DRMs in early intervention settings for Deaf children born into hearing families. Despite the paucity of formalized DRM programs, the reviewed literature highlights many beneficial influences of DRMs for Deaf children that relate to improving a child’s communication, socio-emotional development, and cognition. The five overarching themes that were elicited from the seven reviewed articles were ‘Deaf Gain’ and associated cultural capital, effective communication, developmental influences, family (or caregiver) attitudes to Deafness, and administration of DRM programs. Further research is warranted to provide a holistic analysis that aims to fill a gap in the literature by exploring DRM support within the family context, by exploring the influences from the reflective perspectives of Deaf adults who grew up within a hearing family, and from the perspective of the hearing parents of young Deaf children. Furthermore, it is recommended that an exploration of global best practice be undertaken to investigate the successful international structured role-model programs discussed within the literature. The comparison of practice and policy globally to that which occurs within Australia could lead to the formation of recommendations for the application of a formalized DRM program within Australia.

## Footnotes

1 The capitalization of the word “Deaf” is used intentionally within this paper as an inclusive term that incorporates both the audiological and cultural-linguistic identity aspects of Deafness.

## Appendix

**Appendix A:** Summary of Included Articles

Citation	Country	Aim/Purpose of Study	Participants	Methodology/Methods	Key Findings related to Scoping Review Question	Recommendations and additional comments.
Fobi et al. (2022)	Africa (Ghana)	To examine the development of Early Care and Education (ECE) of Deaf children, and to specifically seek the perceptions of Deaf adults (leaders and advocates) in the development and delivery of early childhood education programs for Deaf children	17 Deaf adults with a range of leadership and advocacy roles	Qualitative Interviews and thematic analysis	Deaf adults prioritize use of sign language for communication. When parents learn sign language, many communication concerns caused by language barriers are mitigated as children are not deprived of an accessible language. Access to Deaf mentors and sign language tuition can be limited. Training and structure for DRMs provides enormous benefits for Deaf children and their hearing family members, and thus efforts for expanding this into the ECE field are important. So too are collaborations with available services that support Deaf students. All participants welcomed the idea of the participation of Deaf Adults as role models for Deaf children in hearing families, and/or offering workshops and seminars for parents of Deaf children. Identified benefits included language and communication support, building the confidence of the hearing parents, and assurances about the children’s future.	Deaf adults providing multimodal support is important and necessary (e.g., internet, books, sign language apps), especially prior to schooling, however difficult due to funding limits. Deaf adults in leadership within ECE positions are necessary, especially for Deaf children and their hearing families. They can introduce family members to the Deaf community and provide a model of what is possible for Deaf people, thus building confidence of caregivers. Deaf children whose families received DRM support made more language gains, had improved social and connections, and had an improved quality of life compared to those without DRM support. Additionally, the provision of love and warmth by DRM assisted families with acceptance of their child and connection to other services to support the Deaf child.
Gale et al. (2021)	International (North America, Europe, Australia, Asia, Africa, South America)	To explore the supports and the role(s) of Deaf adults working with young Deaf children and their families in family-centered early intervention (FCEI) services, and to investigate how Deaf adults are infused in FCEI around the world	48 survey respondents from six continents, including educators, audiologists, speech pathologists and “other”	Mixed methodology Online exploratory survey	Most respondents reported that their first point of contact with a professional post identification of Deafness in their child was a hearing professional. The majority also responded to say that they were not exposed to a diverse range of Deaf professionals. Reasons for limited number of Deaf professionals included lack of funding, lack of available Deaf people to fulfill such roles and limited encouragement for Deaf people to work in the FCEI field. The most common role for Deaf professionals in FCEI was that of mentor/role model or sign language instructor. Deaf professionals mainly provided support to in the areas of educational information and communication support.	The authors recommend Deaf adults be connected with families, infused in FCEI as most hearing parents have minimal experience with Deaf people, and consequently have limited expectations for their Deaf child. When discussing results, the authors note that the limited diversity of Deaf professional roles in early intervention services is not reflective of the Deaf community. They hypothesize that the reflected low diversity is related to a limited understanding of the positive impact that DRMS can have in the system. They suggest that framing Deafness as a cultural linguistic identity rather than a pathological condition may address the low number of Deaf professionals working in FCEI. Deaf professionals have a positive impact on children’s development, and infusing Deaf professionals in FCEI requires education, collaboration and formalization.
Giaouri et al. (2022)	Greece Bulgaria Malta	To investigate the involvement of Deaf practitioners as DRMs or sign language teachers, as well as their training for supporting hearing parents of Deaf children and hearing practitioners	36 adults (Deaf and hearing, including practitioners and parents of Deaf children)	Mixed methodology Ongoing multilingual survey	The teaching of sign language by Deaf adults to hearing parents of Deaf children, and to hearing practitioners involved in early intervention for Deaf children varies across different countries, yet many participants agreed that it was important to assist in developing communication skills. Most participants required proficient signing competence from the teachers. Many Deaf adults are not specifically trained as DRMs, rather they are in the position of being sign language teachers (albeit, untrained). The findings demonstrated that there were no specific qualifications available to support or professionally recognise DRM work. Participants noted that training was a highly regarded attribute of a DRM. Participants reported that sign language resources (dictionaries, books, published materials) are not often publicly available. Participants highlighted that Deaf role models need to teach sign language to parents of Deaf children for communication skill development, share knowledge of the Deaf community and provide information on the range of communication approaches.	The lack of communicative competence is recognized as the root cause of most problems that arise when a Deaf child only has exposure to hearing parents, teachers, and professionals—therefore DRMs are necessary to intervene to address language delays. Further research into the benefits of Deaf role models for Deaf children, especially focusing on the children’s psycho-social and language development is recommended.
Golos et al. (2018)	USA	To investigate the extent to which Early Childhood (EC) settings for Deaf children aligned with a more cultural or pathological perspective of Deafness	62 EC educators of Deaf children	Quantitative Survey	Symbolic role models – in books, media etc often presented with a pathological perspective. This did vary with a correlation between EC settings with proficient signers having less pathological material compared with settings with less skilled signers having materials that were more pathologically focused. Deaf EC educators were more bilingual (than hearing EC educators) in the presentation of literacy materials, translating stories to American sign language (ASL), whilst keeping the English text visible. DRMs visiting EC settings was not a regular occurrence for many of the respondents, however, when DRMs were involved in the EC setting, the signing skills of EC teachers of Deaf children was heightened.	DRMs are critical to a child’s development of identity, and linguistic identity is closely tied in, thus accessibility to sign language is important. DRMs providing a cultural perspective of Deafness, and accessible language modeling, are important to allay internalized audist perspectives and delays in literacy and other academic skills later in life for Deaf children. EC classrooms that align with a cultural perspective of Deafness can promote language and social–emotional skill modeling. EC settings need greater access to a diverse range of actual DRMs and symbolic DRMs portraying a cultural perspective of Deafness for the future language, literacy and social–emotional success of Deaf children.
Hamilton and Clark (2020)	USA	To investigate the perceptions of hearing families of a Deaf child who had a Deaf mentor to determine if a DRM eased their concerns about raising a Deaf child	3 families	Qualitative Semi-structured interviews	Families supported by the Deaf Mentor Program developed the confidence that their Deaf child would have positive life outcomes. Parents developed higher expectations for their Deaf children, and an understanding that their child was “different”, not “disabled”. They felt that through the program, they had developed skills to assist their Deaf child navigate life in a predominately “hearing world”. Families understood the importance of learning about Deaf culture and history from a DRM with lived experience of Deafness. DRMs provided a positive image of Deaf people and provided inspiration to the Deaf child and their hearing family. Providing signing language models at point of diagnosis gives child opportunity to develop age-appropriate language development, as auditory access to spoken language may be delayed or not possible. Signing can also provide support for developing spoken language. The provision of a language rich environment is not limited to academic situations but is just as important in the home and family environment. Parents were surprised at how access to a visual language aided in their child’s spoken language development too.	Hearing families are usually presented with information from hearing medical practitioners, not Deaf people. Consequently, there is a risk of language, academic and social delays. DRMs provide cultural capital, language modeling and connection to the Deaf community.
Rogers and Young (2011)	UK	To seek the DRM perspective as part of an evaluation study of the National Deaf Children’s Society Deaf role model project	19 trained Deaf role model/mentors	Qualitative Focus groups	The training of the DRMs highlighted to the participants the importance of a wide variety of DRMs—valuable for families/Deaf children, but also for DRMs themselves to broaden own perspectives. Deaf children with DRMs had increased self-esteem, and their hearing caregivers developed their confidence that their Deaf child could be successful in life. The value of the lived experience of Deafness is not to be underestimated. Young Deaf children learned of practical and technological supports that aided in developing independence (vibrating alarms, captioning, relay services etc). The benefits from having DRMs (e.g., social capital, improved QoL) extends to families, Deaf children, the other professionals who work with them and the DRM themselves. Role model status may be formalized/deliberately created via specific training and job descriptions or may be informal or perceived/attributed through circumstance.	Professionals in the field of Deafness will often provide information and advice, as well as share their own values and expertise, however, it is preferential to have Deaf role models involved with families to share insights into personal experiences of being a Deaf person and what a Deaf person can achieve.
Shuler-Krause and White (2019)	USA	To review available data on DRMs in Early Hearing Detection and Intervention (EHDI) services to determine the availability of DRMs for Deaf children, to explore how families feel about DRM services, and describe the programs that exist to provide DRM services	One survey included coordinators of 22 state-based EHDI services One survey included 303 families with 2–6-year-old Deaf children A third survey included 458 families of 0-6yo Deaf children including information regarding interaction with DRMs	Mixed methodology Review of available data from 3 separate national surveys	Many parents of Deaf children desired a connection with a DRM but found access to such was difficult due to limited and underfunded services. The most frequently reported benefit was DRMs providing information regarding accessible communication—and assisting families in their confidence regarding being able to communicate with their Deaf child. Majority of families who access DRMs have Deaf children aged between 1–2 years old and interactions with DRMs were weekly. Many findings echoed Watkins et al. (from 1998) that benefits included increased parental confidence regarding communication with their Deaf child, increased understanding of Deaf culture/community, and higher expectations for their child’s future.	There is a widespread consensus regarding the positive benefits of DRMs for families of Deaf children, however, there is little data available on this topic. More research must be undertaken to ensure that DRM programs can be supported to provide this beneficial resource to more families.
