# Correction to: Deaf role-models for Deaf children in hearing families: a scoping review

**DOI:** 10.1093/jdsade/enae040

**Published:** 2024-09-12

This is a correction to: Angela Joy, Susan Ledger, Jill Duncan, Deaf role-models for Deaf children in hearing families: a scoping review, *The Journal of Deaf Studies and Deaf Education*, 2024, enae028, https://doi.org/10.1093/jdsade/enae028

In the section entitled “**Deaf Gain**” **and Associated Cultural Capital** of this paper, the following sentence has been corrected, in order to provide clarification of the origin of the term “*Deaf Gain*”:

*Deaf Gain* is a term that Hamilton and Clark (2020) use as an antonym to *hearing loss*, referring to Deaf people as having a “cultural and linguistic difference” rather than being “defective, and needing to be fixed (p. 714).

The updated sentence reads as follows:

*Deaf Gain* is an affirming concept devised by Bauman and Murray (2014) that emphasizes the unique benefits of Deafness, challenging the polarized view of Deafness as a disability. Hamilton and Clark (2020) also use this term as an antonym for *hearing loss*, referring to Deaf people as having a “cultural and linguistic difference” rather than being “defective, and needing to be fixed” (p. 714).

